# Malocclusions and oral dysfunctions: A comprehensive epidemiological study on 359 schoolchildren in France

**DOI:** 10.1002/cre2.719

**Published:** 2023-03-19

**Authors:** Leslie Borsa, Déborah Estève, Carole Charavet, Laurence Lupi

**Affiliations:** ^1^ UFR Odontologie Université Côte d'Azur Nice France; ^2^ Pôle Odontologie, Centre Hospitalier Universitaire Université Côte d'Azur Nice France; ^3^ Laboratoire Microbiologie Orale Immunothérapie et Santé MICORALIS (UPR 7354), UFR Odontologie Université Côte d'Azur Nice France

**Keywords:** dysfunctions, epidemiological study, malocclusions, risk factors

## Abstract

**Objectives:**

The purpose was to conduct a comprehensive study of malocclusions and oral dysfunctions on 11‐year‐old children and to study the risk factors associated with malocclusions.

**Material and Methods:**

A cross‐sectional descriptive epidemiological survey was conducted among 359 children in France. A clinical examination was conducted, and orthodontic and oral functional data were collected. In addition, the need for orthodontic treatment was evaluated using the criteria defined by of the French National Authority for Health (HAS). Finally, a univariate and multivariate analysis was performed to assess the risks associated with malocclusions.

**Results:**

Most children (88%) exhibited a malocclusion, regardless of gender (*p* = .912). The examination of oral functions identified a large number of swallowing (87%) and respiration (42.7%) disorders. The presence of malocclusion was statistically linked to the low position of the tongue at rest (*p* < .001), abnormal swallowing (*p* = .03), and improper mouth breathing (*p* = .001). After a multivariate analysis, the type of respiration (odds ratio [OR] = 3.2 [1.4–7.3]) and the position of tongue at rest (OR = 3.43 [1.7–7.1]) were the two most prominent factors in the prediction of emerging malocclusion.

**Conclusion:**

This epidemiological survey reveals a high prevalence of dental malocclusions and functional disorders. Oral respiration and the low position of the tongue at rest are the most important factors in the prediction of a malocclusion.

## 1 INTRODUCTION

Many authors have shown interest in the impact of dysfunctions on the morphogenesis arch (Delaire, 1997; Grabowski et al., 2007; Phillippe, 2007; Talmant & Deniaud, 2000). In the sixties, Melvin Moss put forward his functional matrix theory, stating “a function to each organ” or better, “each organ to its own function” (Moss & Salentijn, 1969). Since then, his ideas inspired ardent supporters and fervent opponents. Indeed, beside this functional current, appeared the genetic current (Tweed, 1966), and the synthetic one (Björk, 1947; Delaire et al., 1981), which seems to be a compromise between the two latters (Saccomanno et al., 2012). Thus, the Schwartz, for muscular types (Schwartz, 1956) and Sassouni classifications, for skeletal types, (Sassouni, 1969) based on muscle physiology, can really be considered as applications of Moss's theory.

Current data allow us to say that most cranial facial functions are instrumental in developing the face and in establishing occlusion: a balance between the different muscle groups will allow a more harmonious development (Talmant & Deniaud, 2000). Any imbalance and dysfunction will have an impact of morphogenesis and could lead to osseous deformations and anomalies of tooth position, form, and function being closely linked (Ovsenik et al., 2007). Though, the prevalence of oral respiration varies according to the different authors from 15% to 55% (Abreu et al., 2008; De Menezes et al., 2006; Huber & Reynolds, 1946; Leal et al., 2016). The prevalence of primary deglutition also varies from one author to another, reaching 36% for Garliner (1981). However, a very small number of national or international studies have been carried out on this subject. Indeed, the literature review reveals a significant number of “authors’ views” (Delaire, 1997; Grabowski et al., 2007; Phillippe, 2007; Talmant & Deniaud, 2000) without any scientific evidence backed up by a strictly carried out study coming to light; indeed, only one study was identified, carried out in 2006 by the Epidemiology department of the Faculty of Dentistry in Paris (Souames et al., 2006), whose objective was to study orthodontic treatment need in French schools in the Val d'Oise department. It would therefore be interesting to study oral dysfunctions, especially primary swallowing and oral respiration and their consequences on dysmorphy.

Thus, the objective of this epidemiological survey, based on 11 year old patients, was to evaluate, in a screening situation, the relationship between malocclusion (simple extra‐ and intraoral examination) and oral dysfunctions through the low resting position of the tongue, swallowing and respiration and to highlight the potential impact of these risk factors on the development of malocclusions, as well as the need for orthodontic treatment according to the criteria defined by the French National Authority for Health (HAS) (HAS/ANAES et al., 2012). The second objective was to relate all these variables by uni‐ and multivariate analysis to deduce the risk factors associated with malocclusions.

## 2 MATERIAL AND METHODS

### 2.1 Ethical consideration and registration

This study was conducted in accordance with the Declaration of Helsinki. An ethical approval was sought before the study from the internal ethics committee of the Nice University, and an opinion on the project was also sought from the Institutional Review Board of the Nice University following its creation in 2021. It has also issued a favorable opinion on the project (01/2022, number 2022‐008). An authorization to conduct this study was obtained from school authorities, both the senator mayor of the city and the rector of the Academy of Nice approved the protocol, and an agreement was signed between the city of Cagnes‐sur‐mer and the University. The children's parents were requested to sign an informed consent form, after being informed of the purpose and benefits of the study, before the beginning of the study.

This study is registered in the database for registration of clinical studies (ClinicalTrial.gov, identification number NCT04869839).

### 2.2 Study design

This manuscript was written following the CONSORT (Consolidated Standards of Reporting Trials) guidelines.

The study was designed as an exhaustive cross‐sectional study in the sixth‐grade classes of all the elementary schools of the city of Cagnes‐sur‐mer (town of about 50,000 habitants located in the department of Alpes‐Maritimes, France).

### 2.3 Participants, eligibility and setting

A total of 359 children were therefore enrolled in the study among the 416 children registered in six‐grade classes in Cagnes‐sur‐mer, that is, 86% of children, between April and May 2017; Very few parents refused to give their agreement (2%), and the remaining 12% consisted in the children who were absent on the day of the examination.

### 2.4 Inclusion/exclusion criteria

All children whose parents gave their informed consent were included. No exclusion criteria were to be applied.

### 2.5 Dental examinations

Children were welcomed in small groups to learn about oral health care, traumas and nutrition. Children were invited to sit, each in turn, on a chair in a separate room of their classroom to respect the children's privacy and the confidentiality of the collected data. Clinical examinations were carried out using disposable dental kits (probe and mirror) under natural light or artificial light when natural light alone was deemed insufficient, by using a headlamp. Even though the screening was not performed under the same conditions as a dental chairside examination, a ruler was used, and so was a headlamp and a portable go‐kart with an associated compressor and air/water syringe.

All orthodontic examinations were carried out by an orthodontist (ED) and who is an author of the article. The self‐calibration was done beforehand by training with photographs. The other oral examinations were carried out by a general practicionner (LB), who is also an author of the article. Eight students in their final year of study were also present to assist and perform preventive actions with the children.

Each parent received a letter notifying them of their child's oral health status and whether or not an appointment with a dentist or an orthodontist was necessary.

### 2.6 Data collection

The following data were collected:

– Untreated dental caries.
– The Silness and Loë's (1967) plaque index (0: no plaque, 1: plaque not visible to the naked eye but removable with the probe; 2: plaque visible to the naked eye; 3: abundant plaque visible to the naked eye in the sulcus and on the marginal gingiva) (Abreu et al., 2008).
– A extraoral examination: sub‐nasal profile, the nasolabial angle and the labiomental fold; Cephalometric standards for the skin profile were established by Tweed, Steiner, Burstone, Ricketts, Holdaway and Merrifield in the 1960s.

The sub‐nasal profile is assessed in relation to two lines perpendicular to the Frankfurt plane: Izard's plane and Simon's plane.

If the chin point is located:

– In front of Izard's plane: the profile is convexed.
– Behind Simon's plane: the profile is concave.
– Between the two: the profile is normal.

The nasolabial angle is measured between the columella of the nose and the upper lip. The angle should range between 85 and 105 degrees and is influenced by the position and angle of the upper incisors and the anatomy of the nasal columella.

The labiomental fold divides the chin into an upper and lower half.

– A intraoral examination: Angle's classification of molar and canine relationships, increased overjet (overjet>3 mm), presence of a crossbite (yes/no), shifted midlines (yes/no), presence of deep bite (overbite>3 mm) and presence of an open bite (overbite<0).
– A functional examination: type of respiration, type of swallow and position of the tongue at rest.

The modes of breathing and swallowing were registered during clinical examination. The child was observed in a relaxed position. We evaluated respiration by first looking for adenoid facies characteristics: an elongated face, the presence of under‐eye shadows, a pinched nose, parched lips, half‐opened mouth and opened gonial angle. It was noted whether he or she had competent lip closure. Then, as recommended by Villa et al., the Glatzel test (with a dental mirror) and the Rosenthal test were carried out. Every child was asked to breathe with his/her mouth closed. Within 15 breaths, it was stated whether the child needed to open his mouth or if he was out of breath at the end of the test.

Tongue thrust swallowing data was obtained at the time of the clinical examination. To examine the presence of tongue thrusting, the patients were asked to swallow their saliva three times during the same visit. There should be no facial contraction or lingual interposition, and the arches should remain in occlusion. When in doubt, another swallow was requested until the orthodontist was satisfied with their judgment. Tongue thrust was defined as a protrusion of the tongue between the upper and lower incisors or the cuspids during swallowing.

The position of the tongue at rest was estimated by opening the cheek and looking underneath the tongue. We used the criteria of Van Dyck et al. The “physiological” (normal) resting position of the tongue was defined as the tongue in contact with the palate extending to the palatal aspect of the alveolar ridge (and not between the anterior and/or posterior teeth or directed towards the lower anterior teeth).

In addition, for each child included in this survey, an evaluation of the need for orthodontic treatment according to the criteria retained by the HAS was performed (HAS/ANAES et al., 2012). The HAS allows to give some orientations in the framework of a screening, by defining semiological elements which, during a screening, will direct towards a specialized consultation. Any dysfunction can be considered as a warning sign and should lead to a morphological examination. Respiration, swallowing, phonation, mastication, suction or mandibular kinematics should be monitored. The morphological examination includes an exobuccal examination looking for asymmetries, vertical disproportions of the face, alterations of the profile, permanent resting labial inocclusions, alterations of the smile, and an endobuccal examination which observes discordances of the maxillary and mandibular arches, anomalies of the incisal relationships and disturbances of the alignment of the teeth.

In such “screening” conditions in the schools, casts and X rays were not available. We were forced to limit ourselves to clinical examinations.

### 2.7 Sample size calculation and statistical analysis

The previous study carried out in Paris revealed that 20% of the examined children needed and orthodontic treatment (De Menezes et al., 2006). Therefore, the minimum size of the sample to be obtained can be calculated thanks to the following formula:

$$n=\frac{t^{2}\;xp(1-p)}{e^{2}}.$$

With *t* = standard normal variate (at 5% type I error (*p* < .05) it is 1.96).

*p* = expected proportion in population based on the prior study.

*e* = absolute error or precision, set at 5%.

Therefore, the number of children to be included must be of at least 244.

Descriptive statistics were used to summarize data, then frequency tables and univariate analyses (cross‐tabulated) were carried out using the “presence of a malocclusion” as the explained variable. The chi‐squared test or Fisher's exact test were used for qualitative variables, whereas the Student *t* test was used for quantitative variables. A significance threshold of .05 was adopted. The multivariate analysis, using a binary logistic regression, was carried out by inserting all the 0.20 univariate significant variables into the model. The software used was SPSS 25.0.

## 3 RESULTS

### 3.1 General characteristics of the studied population

A total of 416 children attended six‐grade classes in the public schools of the city of Cagnes‐sur‐mer. Among them, nine could not be examined because their consents were not obtained, and 48 were absent on the day of the study. The present study therefore examined 359 children, the study participation rate reaching 86%. The average age was 10.98 ± 0.43 years. The gender‐ratio was 0.48 (Table 1). The schoolchildren were quite evenly split between the different neighborhoods of the city. No children with syndromes or disability was included in the study, as the study was led in a primary school, not in a Medico Educational Institute.

**Table 1:** General characteristics of study population.

	Characteristics	%
Gender	Boy	48
	Girl	52
Age group	10–11 years old	3.9
	11–12 years old	87.7
	12–13 years old	8.4

### 3.2 Oral data

#### 3.2.1 Dental status

The children had an overall satisfactory oral health status. More than two‐thirds (71.5%) had never had a dental decay, and only 10.6% had ever received conservative care. However, 12.6% exhibited untreated dental cavities on their permanent teeth. Based on the Silness and Loë's (1967) plaque index, nearly half of all schoolchildren showed either plaque, which could be seen with the naked eye (35.2%), or an abundance of soft matter (12.3%). Only one out of 5 children (27.1%) had no plaque at all. About 20% of all children were engaged in active orthodontic treatment.

#### 3.2.2 The orthodontic examination consisted in

– An extraoral examination of the schoolchildren, which allowed to apprehend the form of the sub‐nasal profile, the majority of which were convex or straight (48.6% and 47.5%), concave profiles being rare (3.9%). The nasolabial angle was very often normal (60.6%) and opened in 24.9% of cases. The labiomental fold was normal (60.6%) or jutting (27.7%) and rarely faded (11.7%).
– The endobuccal examination revealed a canine and molar relationship evenly distributed between Classes I and II while classes III represented only 3% of the children. It also revealed an absence of crossbite in 87.7% of the cases and a bilateral crossbite in only 2.2% of the children. However, more than one‐third of the children had a shifted midline (33.2%). An increased overjet was present in half of the cases (50.3%), and the presence of a deep bite was found in 44.7% of the cases In contrast, the presence of an open bite was infrequent (about 5%) (Table 2).
– The examination of oral functions revealed that atypical swallowing was found in most children (87%), nasal respiration was present in only half of the children, and the resting tongue position was low in most cases (85%) (Table 3).

**Table 2:** Dental characteristics of study population.

	Characteristics	%
Right molar class	1	48.6
	2	47.8
	3	3.6
Left molar class	1	43.9
	2	53.1
	3	3.1
Shifted midlines	Yes	66.8
	No	33.2
Cross‐bite	None	87.7
	Right	4.2
	Left	5.9
	Bilateral	2.2
Overjet > 3 mm	No	49.7
	Yes	50.3
Deep bite	Present	44.7
	Absent	55.3
Open bite	Present	4.7
	Absent	95.3

**Table 3:** Functional characteristics of study population.

	Characteristics	%
Respiration	Nasal	50.3
	Oral	42.7
	Mixed	7
Swallowing	Normal	12.6
	Primary	87.4
Tongue position at rest	Normal	15.4
	Low	84.6

Additionally, 88% of children needed an orthodontic treatment, according to the HAS classification.

### 3.3 Univariate analyses exploring the relationships between the need of orthodontic treatment and other variables

Overall, most children (88%) exhibited a malocclusion, regardless of gender (*p* = .912) or age (*p* = .18). At a functional level, malocclusions were statistically linked to a low position of the tongue (*p* < .001), a primary swallowing (*p* = .03) and a mixed or oral respiration (*p* = .001; Table 4).

### 3.4 Multivariate analysis

Finally, to prioritize the effects of univariate significant variables on our variable of interest, a logistic regression was carried out. After completing the multivariate analysis, only two variables remained significant, the others disappearing to their benefit: an abnormal respiration (mixed or oral) and a low position of tongue at rest, have a threefold increase in risk for malocclusion (Table 4).

**Table 4:** Uni and multivariate statistical analysis.

		Orthodontic treatment need (*n*)		Univariate analysis *p*	Multivariate analysis odds ratio (95% confidence range)
		Yes	No		
Gender	Girls	164	22	.52	Not significant
	Boys	151	21		
Age	10–11	11	3	.18	Not significant
	11–12	275	39		
	12–13	29	1		
Presence of plaque	None	75	22	.002*	Not significant
	Few	84	7		
	Visible	115	11		
	Abundant	41	3		
Subnasal profile	Right	140	30	.008*	Not included
	Convex	162	12		
	Concave	13	1		
Labiomental fold	Normal	185	32	.088	Not included
	Prominent	93	6		
	Not very prominent	37	5		
Nasolabial angle	Normal	186	31	.19	Not included
	Closed	49	3		
	Opened	80	9		
Right molar angle class	Cl I	133	41	<.0001*	Not included
	Cl II	169	2		
	Cl III	13	0		
Left molar angle class	Cl I	116	41	<.0001*	Not included
	Cl II	188	2		
	Cl III	11	0		
Shifted midlines	Yes	197	42	<.0001*	Not included
	No	118	1		
Overjet > 3 mm	Normal	138	40	<.0001*	Not included
	Increased	177	3		
Cross bite	None	271	43	.26	Not included
	Right	15	0		
	Left	6	0		
	Bilateral	8	0		
	Post	15	0		
Deep bite	Yes	159	1	<.0001*	Not included
	No	156	42		
Open bite	Yes	17	0	.241	Not included
	No	298	43		
Respiration	Nasal	148	32	.001*	3.2 [1.4–7.3]*
	Mixed	22	3		
	Oral	145	8		
Swallowing	Normal	35	10	.03*	Not significant (0.63)
	Primary	280	33		
Position of tongue at rest	Normal	39	16	<.0001*	3.43 [1.7–7.1]*
	Low	276	27		

## 4 DISCUSSION

According to the present epidemiological survey of 359 children, the prevalence of dental malocclusions and functional disorders is significant, associated with a high need for orthodontic treatment according to HAS criteria. Furthermore, oral respiration and low tongue position at rest are the most important factors in the prediction of malocclusion, thus encouraging the prevention and correction of functional disorders as soon as possible.

This cross‐sectional descriptive epidemiological survey included 359 children, 86% participation rate, which confirms the good representativeness of our sample, in agreement with our power calculation. Fifth‐grade children (mean age 11 years) were selected, in accordance with the age reference chosen by the World Health Organization (WHO). Moreover, This age corresponds to the last year of primary school, which seems to us to be the right time to carry out a screening and refer to an orthodontist if this is necessary and the child is not already being treated. The variable of need for orthodontic treatment was defined according to the criteria selected by the HAS, which remains the French reference since 2012.

### 4.1 Need of orthodontic treatment

Overall, among the 359 examined children, 88% exhibited a malocclusion. This figure is bigger than the one found in Souames et al. study, conducted in 12 schools in the Val d'Oise department in 2006 (Souames et al., 2006). In this study, 28.6% of children were on the brink of an orthodontic treatment need, and 21.3% really needed treatment. However, these results seem to be difficult to compare with the result of this present study because the index measuring the need for treatment is different. The index used by Souames et al. was the Index of Orthodontic Treatment Need (IOTN), while the HAS recommendations were not in force yet in France. In 1989, a study conducted by Brook and Shaw (1989) in Manchester showed that 32.7% of 11–12‐year‐old schoolchildren needed orthodontic treatment whereas the study conducted by Burden in Ireland in 1995 showed that 36% were in need of orthodontic treatment (Burden, 1995). However, Ingervall and Hedegard noted 53% of care needs in 1975 in Lapland (Ingervall & Hedegård, 1974). Therefore, results vary both according to the country where the study is conducted and the index chosen.

### 4.2 Caries status

The dental health status of the examined children was rather satisfactory since 71.5% of them had never experienced dental caries. The number of untreated decay has clearly improved compared to the national figures obtained during the last survey, conducted by the French Union for Oral Health UFSBD (Union Française pour la Santé Bucco‐Dentaire, 2012) in 2006, where only 56% of children were caries‐free (HAS/ANAES et al., 2012). It therefore seems that the prevention campaigns conducted for many years in France were successful. However, the key factor of this success lies probably in the fact that the Sud région in general, and the Alpes‐Maritimes department in particular, benefit from a very high standard of living, which is known to be highly correlated to dental health status (Muller‐Bolla et al., 2015).

### 4.3 Part played by oral functions

Nearly half of the examined children had abnormal respiration. Under normal circumstances, and particularly at rest, in healthy subjects, the only physiological respiratory route is the nasal passage. Oral respiration is a complement used when highly needed (physical activity, stress…) or when there is an obvious nasal obstruction. The existence of labial open bite is considered as the translation of oral or mixed respiration (Harari et al., 2010). This is the reason why, in conducting the functional examination, the respiration was defined as “abnormal” when spontaneous opening of the lips was seen during observing respiration. Oral respiration is, therefore pathological: the mouth being open, this induces a low position of the tongue, which leads to mandibular propulsion, hypo development of the upper jaw as well as hyper divergence and open bite (Rossi et al., 2015). Patients present an “adenoid” facial appearance: elongated face, opened mouth, pinched nose, under‐eye shadows, and dry lips (Raffat & Ul Hamid, 2009). Overall, sleep disorders with non‐restorative sleep and snores is frequently related with these malocclusions (Katyal et al., 2013). In this study, the high percentage of oral respiration could also be explained by the season in which the study was carried out. Indeed, at the end of spring and at the beginning of summer in Provence, many allergies occur due to an important exposure to pollens, particularly those of olive trees and cypresses.

Regarding swallowing, more than 87% of children experienced abnormal swallowing in this study. Regarding swallowing, more than 87% of children showed abnormal swallowing in this study. This figure seems high, although in the literature, not all authors are unanimous about the frequency of dysfunctional swallowing. Indeed, the prevalence of primary swallowing varies from 39% for Hanson and Cohen (1973) to 75% for Launey et al. (2014). For Fletcher et al. (1961), atypical swallowing decreases with age. Moreover, the Hanson report on the prevalence of lingual propulsion depending on age also shows a decrease in lingual propulsion in mixed dentition: it would be present in 40%–50% of children in early mixed dentition at 6/7 years of age, then it would decrease to nothing in 30%–40% in late mixed dentition at 11/12 years of age (Hanson & Cohen, 1973). A good position of the tongue is important because it is maintained about 22 h a day. However, in the case of oral respiration, the tongue is in low position to disengage the upper airway (Garliner, 1981).

### 4.4 Interactions between variables and the need for orthodontic treatment

Furthermore, this study not only aimed to estimate the oral health status and orthodontic need of children in a city in France: the objective was also to see to what extent the need for orthodontic treatment was correlated with oral dysfunction.

In the multivariate analysis conducted in this study, there were two significant variables: abnormal respiration and low position of tongue. Indeed, the analysis shows that when there is abnormal respiration, the probability of needing orthodontic treatment is multiplied by approximately 3 (OR = 3.2 [1.4–7.3]_95%_). This probability is multiplied by 3.43 when the tongue is in a low position at rest (OR = 3,43 [1.7–7.1]_95%_). In agreement with the literature cited in the previous paragraph and the high rate of need for treatment found in this study, low tongue position and dysfunctional respirations are the most influential criteria regarding alveolodental dysmorphosis. It would appear that low tongue position and dysfunctional repirations are the most influential criteria regarding alveolodental dysmorphosis, but other factors not investigated in this study and worthy of further study are also to be considered, such as the impact of growth. This is in agreement with Frapier et al. (2015) who showed in 2005 the outbreak of dysmorphy related to dysfunctions. However, the presence of a primary swallow was not significantly correlated with the need for orthodontic treatment (*p* = .63). These results are in agreement with the conclusions of other authors who believe that lingual propulsion during swallowing is not the cause of malocclusions (Raffat & Ul Hamid, 2009): the low position of the tongue, due to its period of application (Harari et al., 2010; Raffat & Ul Hamid, 2009), would most likely be the main cause of it.

## 5 LIMITATIONS

The need for orthodontic treatment was evaluated according to the criteria defined by the HAS. It would be advisable to evaluate this need using the IOTN index, which is the world reference index in this area, to be able to compare our results more easily with those of other research teams. This study was conducted in a screening context, and it was therefore not possible to take radiographs or dental impressions. The examinations were carried out with portable equipment, although artificial light was used when natural light alone was deemed insufficient. Indeed, in a screening context, the authorizations given by the official authorities (Hospital and Dental School of Nice) can only concern non‐interventional procedures. Thus not all of the IOTN criteria could be evaluated.

## AUTHOR CONTRIBUTIONS

*Conception and design*: Laurence Lupi. *Acquisition of data*: Leslie Borsa and Déborah Estève. *Analysis and Interpretation of data*: Leslie Borsa, Déborah Estève, Carole Charavet, and Laurence Lupi. *Drafting the manuscript*: Leslie Borsa, Déborah Estève, Carole Charavet, and Laurence Lupi. *Reviewing and editing the manuscript*: Leslie Borsa, Déborah Estève, Carole Charavet, and Laurence Lupi. All authors gave final approval and agreed to be accountable for all aspects of the work.

## CONFLICT OF INTEREST STATEMENT

The authors declare no conflict of interest.

## Data Availability

The data that support the findings of this study are available from the corresponding author upon reasonable request.
